# The Impact of SARS-CoV-2 Pandemic on Patients Undergoing Radiation Therapy for Advanced Cervical Cancer at a Romanian Academic Center: A Four-Year Retrospective Analysis

**DOI:** 10.3390/diagnostics12061488

**Published:** 2022-06-17

**Authors:** Alin Popescu, Stelian Pantea, Daniela Radu, Adrian Gluhovschi, Catalin Dumitru, George Dahma, Adelina Geanina Mocanu, Radu Neamtu, Sorin Dema, Codruta Victoria Tigmeanu, Mirela Loredana Grigoras, Silvius Alexandru Pescariu, Hazzaa Aabed, Marius Craina

**Affiliations:** 1 Department of Obstetrics and Gynecology, “Victor Babes” University of Medicine and Pharmacy Timisoara, 300041 Timisoara, Romania; alinp22@yahoo.com (A.P.); adigluhovschi@yahoo.com (A.G.); dumcatal@yahoo.com (C.D.); george_dahma@yahoo.com (G.D.); adelinaerimescu@yahoo.com (A.G.M.); radu.neamtu@umft.ro (R.N.); mariuscraina@hotmail.com (M.C.); 2 Department of General Surgery, “Victor Babes” University of Medicine and Pharmacy Timisoara, 300041 Timisoara, Romania; pantea.stelian@umft.ro (S.P.); daniela_radu@hotmail.com (D.R.); 3 Discipline of Radiology, “Victor Babes” University of Medicine and Pharmacy Timisoara, Eftimie Murgu Square 2, 300041 Timisoara, Romania; sorindema@yahoo.com; 4 Department of Technology of Materials and Devices in Dental Medicine, Multidisciplinary Center for Research, Evaluation, Diagnosis and Therapies in Oral Medicine, “Victor Babes” University of Medicine and Pharmacy, Eftimie Murgu Square 2, 300041 Timisoara, Romania; 5 Department of Anatomy and Embryology, “Victor Babes” University of Medicine and Pharmacy Timisoara, 300041 Timisoara, Romania; grigoras.mirela@umft.ro; 6 Department VI, Cardiology, “Victor Babes” University of Medicine and Pharmacy, Eftimie Murgu Square 2, 300041 Timisoara, Romania; alex.pescariu@yahoo.com; 7 Department of Plastic Surgery, “Victor Babes” University of Medicine and Pharmacy Timisoara, 300041 Timisoara, Romania; haza_a_dr@hotmail.com

**Keywords:** SARS-CoV-2, COVID-19, cervical cancer, radiotherapy, chemotherapy

## Abstract

*Background and Objectives*: Throughout the COVID-19 pandemic, health systems worldwide adapted to support COVID-19 patients while continuing to provide assistance to patients with other potentially fatal illnesses. While patients with cancer may be at an elevated risk of severe COVID-19-related complications, their oncologic therapies generally cannot be postponed indefinitely without a negative effect on outcomes. Taking this into account, a thorough examination of the therapy management of various cancers is necessary, such as cervical cancer. Therefore, we aimed to develop a retrospective cohort study to measure the impact of the COVID-19 pandemic on the delivery of cancer care services for women diagnosed with cervical cancer staged IB2-IVA, necessitating chemo- and radiotherapy in Romania, as well as determine the difference in cervical cancer staging between the pandemic and pre-pandemic period. *Materials and Methods*: Using a multicentric hospital database, we designed a retrospective study to compare the last 24 months of the pre-pandemic period to the first 24 months of the SARS-CoV-2 pandemic to evaluate the variation in the proportion of women diagnosed with cervical cancer and the percentage of inoperable cases requiring chemotherapy and radiotherapy, as well as to detail their clinical presentation and other findings. *Results*: We observed that the likelihood of cervical cancer patients requiring radiation therapy at a later stage than before the pandemic increased by about 20% during the COVID-19 pandemic. Patients at an advanced FIGO stage of cervical cancer had a 3.39 higher likelihood of disease progression after radiotherapy (CI [2.06–4.21], *p*-value < 0.001), followed by tumor size at diagnosis with a hazard ratio (HR) of 3.12 (CI [2.24–4.00], *p*-value < 0.001). The factors related to the COVID-19 pandemic, postponed treatment and missed appointments, were also identified as significant risk factors for cervical cancer progression (HR = 2.51 and HR = 2.24, respectively). *Conclusions* We predict that there will be a considerable rise in cervical cancer cases over the next several years based on existing data and that expanding screening and treatment capacity will attenuate this with a minimal increase in morbidity and fatality.

## 1. Introduction

Cervical cancer is potentially preventable and even curable in its early stages, but early diagnosis requires a good screening and early detection program, as well as access to suitable therapies when necessary [1,2]. In contrast to many cancers, the natural history of HPV infection and cervical intraepithelial neoplasia is well understood and includes a noninvasive latent phase during which screening and prevention measures can be conducted, accompanied by an early invasive phase during which disease remains localized to the cervix, and therapeutic interventions are typically curative [3,4]. Although brief treatment delays do not result in substantial changes in outcomes, prolonged delays result in disease progression and more significant fertility loss, resulting in a decline in overall survival [5,6,7].

Starting in December 2019, a new coronavirus depicted as the severe acute respiratory syndrome coronavirus 2 (SARS-CoV-2), responsible for the disease called COVID-19, has been spreading rapidly around the globe [8], requiring all nations to cope with the COVID-19 pandemic’s dangers and restrictions [9,10]. Certain types of individuals, such as the elderly, pregnant women, or those with chronic conditions, have a greater risk of negative consequences from SARS-CoV-2 infection [11,12,13,14]. Regarding cancer patients, the available statistics indicate a greater risk of life-threatening infections [15]. The existence of active cancer and its treatment might decrease physical ability and induce immunosuppression, increasing the need for health care visits and hospitalization [16]. All of these conditions may enhance the probability of infection with COVID-19 and the development of negative outcomes [17,18,19].

Throughout the ongoing COVID-19 pandemic, hospitals modified their organizational workflows, reducing staffing and redesignating inpatient beds for COVID-19 care [20,21]. This resulted in decreased staffing and bed capacity for all non-COVID departments, including the availability for cancer patients and their cancer treatment via chemotherapy, brachytherapy, or external radiotherapy [22,23]. In accordance with evolving recommendations for radiation therapy for gynecological cancers during the COVID-19 pandemic, the policy for cancer care management should be reorganized to improve patient treatment and follow-up. However, implementing COVID-19 guidelines presents numerous challenges due to the pandemic’s prolonged restrictions involving close human contact, even while a full-scale vaccination campaign against SARS-CoV-2 is ongoing [24,25].

In countries with a centralized database of cancer patients, it was possible to assess the pandemic effects on the epidemiology of cervical cancer during the pandemic and forecast outcomes based on the missed diagnoses and appointments. In England, the proportion of surplus patients has already been estimated and indicates that up to 630 more cases of invasive cervical cancer might be detected in the following three years [26]. In contrast, apart from some early simulations that speculated on the potential consequences of a lack of diagnostic services, there are limited real-world statistics on the number of concealed invasive cancer during the pandemic. Therefore, the purpose of this research is to provide a series of data and real-world statistics involving women with cervical cancer from Romania during the COVID-19 pandemic. The main focus is on the cases that required radiotherapy or combination with chemotherapy, describing clinical features, cancer diagnosis and progression, and available treatment. The secondary end point is to analyze the outcomes of patients treated in our institute as risk factors for disease progression after radiation therapy.

## 2. Materials and Methods

### 2.1. Background, Design, and Ethics

Observational research was conducted in the University Clinic of Obstetrics and Gynecology “Bega” of the Timis County Emergency Clinical Hospital “Pius Brinzeu” in Timisoara, Romania, which is associated with the “Victor Babes” University of Medicine and Pharmacy. Our research used a retrospective cohort design in partnership with the Timisoara Municipal Emergency Hospital’s Radiology Department. The research population and its relevant features were identified using a population-based administrative database of outpatients and inpatients from the two hospitals involved throughout the study period. Our centralized database contained patient medical records that were protected by privacy laws and obtained with the consent of the patient, including their medical history, cervical cytology tests, and surgical and oncological data. The Ethics Committee of the “Victor Babes” University of Medicine and Pharmacy in Timisoara, Romania, as well as the Ethics Committees of both hospitals, accepted the study protocol.

### 2.2. Inclusion Criteria, Patient Characteristics, and Study Variables

Between January 2018 and January 2022, the study included adult women older than 18 who presented for cancer treatment after having a confirmed cervical cancer diagnosis based on cervical screening cytology, colposcopy, and other invasive methods with biopsy, using conventional methods [27,28]. The research did not follow a certain sampling method and included all consecutive patients scheduled for radiation therapy or combined treatment for cervical cancer, as well as those planned for regular follow-up at the two hospitals’ gynecologic oncology units if they matched the inclusion criteria. Those patients whose tests and diagnoses were not verified and who lacked necessary information or permission to participate in the present investigation were excluded. Another exclusion criterion comprised the participants that have been lost to follow-up three months after cancer therapy. A total of 104 patients were selected from the pandemic period that were case-matched by age with a group of 104 patients from the pre-pandemic period.

Radiation treatment was considered for patients based on the most recent American Society for Radiation Oncology (ASTRO) recommendations [29]. The guideline advises postsurgical radiation therapy for patients with intermediate-risk factors and chemoradiation for those with high-risk factors, while in the definitive setting, chemoradiation is recommended for stages IB3-IVA based on the International Federation of Gynecology and Obstetrics (FIGO) staging system [30], and radiotherapy or chemoradiation is recommended conditionally for stages IA1-IB2 if they are medically inoperable. This involves the curative treatment of invasive cervix carcinomas such as squamous cell carcinomas and adenocarcinomas. It emphasizes the treatment of cervical cancer using radiation therapy, including the indications, procedures, and results, while also discussing alternative therapies that may alter the effectiveness of radiation therapy when used simultaneously or sequentially with chemotherapy and/or surgery. This research focuses only on patient features and treatment at tertiary-level cancer centers, not on the influence of SARS-CoV-2 infection on admission, investigation, or diagnosis in primary care in conjunction with the presence of malignancy. However, cases of SARS-CoV-2 infection during admission were not excluded, and a positive COVID-19 diagnosis was confirmed by nasopharyngeal RT-PCR detection for SARS-CoV-2 RNA, according to guidelines at the time of the study [31,32].

Representatives of the clinical teams collected data on all cervical cancers diagnosed during the study period that were anonymized prior to analysis. The following variables were gathered: 1. background data and baseline characteristics of the participants (age, body mass index (BMI), smoking history, menopausal status, number of parties, place of origin, occupation, level of income, civil status, SARS-CoV-2 infection); 2. radiotherapy characteristics (radiation therapy type, moderate/severe acute toxicity, moderate/severe late toxicity, response to treatment at three months, referral source, referred and received treatment, changes in the treatment plan, postponed treatment, missed appointments); 3. cervical cancer characteristics (comorbidities, cancer histology, tumor size, invasion of the vagina, parameters, differentiation grade, FIGO stage, radical hysterectomy, cancer relapse status, palliation, reason for palliation, necessity for hospitalization during radiation therapy, the duration of hospitalization). 

### 2.3. Statistical Analysis

The software used for statistical analysis were MS EXCEL and IBM SPSS version 27. Continuous variables were expressed as the mean ± standard deviation (SD) or as the median with interquartile range (IQR). To compute the means and standard deviations, descriptive statistical analyses were conducted, while a Student’s *t*-test was performed to determine the *p*-value. To analyze the differences in proportions, the Chi-square test was utilized. A Cox regression model was built to determine factors that influence disease progression. It was decided that a *p*-value of 0.05 was statistically significant.

## 3. Results

### 3.1. Comparison of Baseline Characteristics

During the study period of 48 months, a total of 208 patients were selected by matching inclusion criteria and case-matching by age, making a group of 104 women with cervical cancer identified in the 24 months before the beginning of the COVID-19 pandemic, and other 104 patients identified during the first 24 months of the pandemic. The average patient was 54 years old, without statistically significant differences in the proportions of body mass index, smoking history, number of parities, place of origin, occupation, level of income, and civil status. As presented in Table 1, more than 30% of the entire cohort of patients comprise smokers, with proportions of women at menopause being around 50% with post-menopausal women. 

A total of 31.7% of the cohort were nulliparous women in before the pandemic and 33.7% during the pandemic (*p*-value = 0.915). A total of 60% all participants were from urban areas, and the majority were employed (61.5% before COVID-19 vs. 52.9% during COVID-19, *p*-value = 0.564). More than 80% of women included in the study were married, and the level of income of the majority was medium, with insignificant differences between study groups (*p*-value = 0.784). 

### 3.2. Comparison of Cervical Cancer Characteristics

Table 2 describes cervical cancer characteristics of women undergoing radiation therapy before and during the COVID-19 pandemic. There was no significant difference observed between the proportion of comorbidities in the groups analyzed before and during the pandemic, where hypertension was the most commonly found—in 80 (38.4%) patients from the full cohort. The cervical cancer histology was squamous cell carcinoma in 168 (80.7%) cases, with no significant differences between the study groups (*p*-value = 0.724). Additionally, the tumor size difference was statistically significant, as 59 (56.7%) tumors identified before the pandemic were smaller than three centimeters in size, compared to 64 (57.7%) of them being bigger than 3 cm in the cohort during the pandemic (*p*-value = 0.037). 

**Figure 1:**
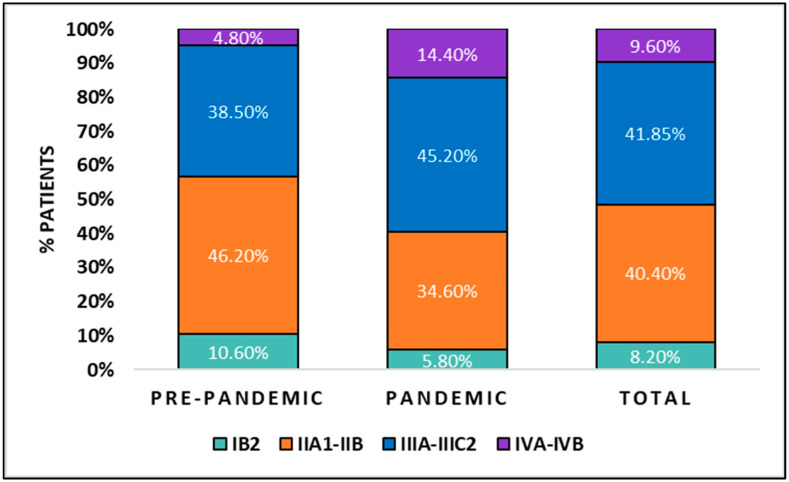
Graphical comparison of patients with radiotherapy-necessitating cervical cancer (IB2-IVB) before and during the COVID-19 pandemic. Cervical cancer staging is reported by International Federation of Gynecology and Obstetrics (FIGO) staging system.

Parametrial invasion and tumor grading did not differ between the study groups, although the invasion of the vagina was significantly more extended in the patients presenting for radiation therapy during the COVID-19 pandemic (24.0% extended to the lower third of the vagina, compared with 12.5% of cases before the pandemic; *p*-value = 0.046). The tumor staging by the FIGO staging system was also notably different between study groups, observing more advanced stages of cancer presenting for treatment during the pandemic (14.4% vs. 4.8% IVA-IVB; *p*-value = 0.032), as well as more cases of relapse (27.9% vs. 16.3%; *p*-value = 0.044) that added to the number of patients treated for palliation (63.5% vs. 48.1%; *p*-value = 0.034) (Figure 1). 

### 3.3. Comparison of Radiotherapy Characteristics

Among the study participants, 82 (78.8%) underwent external beam radiotherapy before the pandemic, compared with 81.7% during the pandemic (*p*-value = 0.601). The most commonly observed acute toxicity was anemia from radiation in 132 (63.4%) patients from the full cohort, followed by leucopenia in 127 (61.0%) patients and skin toxicity in 107 (51.4%) patients with radiation therapy for cervical cancer. The most common late toxicity affected the intestines in 60 (28.8%) cases (Table 3).

Among the significant findings, it was observed that 22.1% of patients during the COVID-19 pandemic had disease progression after completing the radiation therapy regimen, compared with 11.5% of patients before the pandemic (*p*-value = 0.045). The referral sources were mainly from primary care before the pandemic (64.4%) and from secondary care (51.0%) during the pandemic (*p*-value = 0.025). Those who were referred to and did not receive treatment during the pandemic were 13.5% more than before the pandemic (*p*-value = 0.021). There were also significant alterations in treatment outcomes, where 25.0% of patients had changes in their treatment plans during the pandemic, compared with 13.5% before the pandemic (*p*-value = 0.034). A total of 22 (21.2%) patients had postponed treatment and 23.1% missed appointments for various reasons during the pandemic, compared to 9.6% before the pandemic, 12.5% (*p*-value = 0.021 and 0.015, respectively).

### 3.4. Cox Regression Model

**Figure 2:**
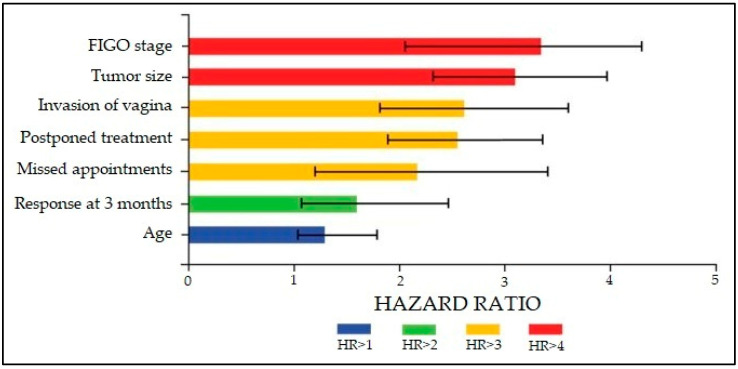
Graphical representation of risk factors for disease progression in patients with cervical cancer undergoing radiation therapy. The likelihood of disease progression reported as hazard ratio (HR) and confidence interval.

A Cox regression model to analyze risk factors for disease progression after finishing the radiation therapy regimen is presented in Table 4 and Figure 2 in descending order of hazard ratios. Patients with an advanced FIGO stage of cervical cancer had a 3.39 higher likelihood of disease progression after radiotherapy (CI [2.06–4.21], *p*-value < 0.001), followed by tumor size with an HR of 3.12 (CI [2.24–4.00], *p*-value < 0.001). The factors related to the COVID-19 pandemic, postponed treatment and missed appointments, were also identified as significant risk factors for cancer progression (HR = 2.51 and HR = 2.24, respectively). Other significant factors were the invasion of the vagina, response to treatment at three months, and patient age.

## 4. Discussion

In the current study, it was demonstrated in a retrospective fashion how the COVID-19 pandemic in Romania influenced cervical cancer diagnosis and management among cervical cancer patients in the advanced and inoperable stages of the disease. These findings corroborate most of the predictions and conjecture suggesting that many cancer cases were missed throughout the ongoing pandemic, as previously observed in the entire population of patients with cervical cancer from Romania during the pandemic [33]. Additionally, we have considered that likely many patients skipped appointments or intentionally delayed or intentionally denied treatment after having a low-stage cervical cancer diagnosis that, although curable in an early phase, became inoperable, necessitating chemotherapy, radiotherapy, or combined therapy. We discovered that the risk of cervical cancer presenting for radiation treatment at a later phase than before the pandemic increased by almost 20%.

Radiation therapy is critical in the management of cervical cancer. In the past twenty years, radiation oncology technologies have advanced rapidly. Particularly, the use of combination immunotherapy with radiotherapy has significantly enhanced treatment results and toxicity profiles for patients with cancer [34], and they are now regarded as the gold standard in many nations, although are still not widely available in Romania. Experimental perspectives include the addition of immunotherapy to chemoradiation regimens or a move toward an even more individualized approach to treatment [35] with the identification of risk factors and biomarkers that can be used to de-escalate or intensify treatments based on the risk group of a particular patient.

A recently published meta-analysis of over 5000 patients determined that adjuvant chemotherapy after radical hysterectomy may be an effective alternative to a combined regimen of chemotherapy and radiotherapy for cervical cancer patients, particularly younger women who wish to preserve their ovaries and prevent radiation damage to them. It significantly decreased the risk of distant recurrence (OR = 0.67), and better rates of overall survival (OR = 0.69), and disease-free survival rate (OR = 0.77) were related to adjuvant treatment [36].

Our patients had similar toxicity profiles after radiation treatment, as reported by other studies [37]. It was observed that among the forty-four patients who received a full course of radiation therapy, 93.2% had a complete response. In general, the medication was well tolerated, and toxicities were within tolerable limits; nevertheless, moderate-to-severe toxicities were seen in the form of anemia and leucopenia. The most prevalent late toxicities involved the small and large intestines. A total of 9.1% of patients had severe (grade 4) late toxicities, including two cases of grade 4 bowel toxicity due to fistula development and one instance of subacute intestinal occlusion. One patient had symptoms of grade 3 bowel toxicity, while another exhibited grade 3 bladder toxicity. In our study, we observed that 63.4% of patients from the full cohort had anemia after radiation treatment, followed by leucopenia in 61.0% of cases and skin toxicity in 51.4% of patients. The most common late toxicity affected the intestines in 28.8% of cases.

The trends in cervical cancer outcomes in Romania might generally follow a negative turn during and after the COVID-19 pandemic, as compared with the period before, described by Furtunescu et al. [38]. Their 20-year epidemiological study indicated significant variations in cervical cancer death rates between Romania and the European Union, as well as within Romania. Despite a 13 percent drop in fatalities and a 25 percent reduction in mortality over the previous two decades, the disparities continue to be rather large. Romania maintains the top spot in the EU in terms of mortality rate, with a significant disparity from the EU average. Despite a greater decline in rural mortality than urban death, the rural–urban mortality disparity reached 24 percent of the national rate in 2019. The same disparities can further deepen during the pandemic, as observed by our findings, leaving a wider gap between Romania and other European countries within the same range. However, with the implementation of the HPV vaccination campaign throughout the European Union, and an increasing trend in its adoption in Romania [39], it is expected for cervical cancer cases to plummet in the future, thus minimizing the impact of a pandemic such as COVID-19.

The current study integrates critical data on the epidemiology, public health, and clinical features of cervical cancer patients from Romania. However, it is limited by the retrospective design relying on strong patient recordkeeping and the quality of data digitally copied from paper records. Another limitation is the sample size that was restricted by our hospital database; therefore, the current results might not confidently represent the characteristics and outcomes of patients with cervical cancer from Romania during the COVID-19 pandemic.

## 5. Conclusions

Although cervical cancer is not among the most common cancers, it is still likely that many cases were undiagnosed during the COVID-19 pandemic, and failure to do so will have long-lasting consequences if these patients are not discovered and treated promptly. A comprehensive cervical cancer screening campaign is justified after the pandemic restrictions are lifted, as well as for the other common cancers detectable by screening methods. Future directions should focus on a prospective analysis and follow-up of existing patients diagnosed during the COVID-19 pandemic to validate the forecasted predictions and estimate with greater accuracy the pandemic effects on patients with cervical cancer.

## Figures and Tables

**Table 1:** Comparison of baseline characteristics of women with cervical cancer undergoing radiation therapy before and during the COVID-19 pandemic.

	Before COVID-19 (*n* = 104)	During COVID-19 (*n* = 104)	*p*-Value *
**Background**			
Age, years (mean ± SD)	54.1 ± 16.1	54.0 ± 16.6	0.965 **
BMI, kg/m^2^ (mean ± SD)	25.8 ± 3.6	26.4 ± 3.3	0.211 **
Smoking history	36 (34.6%)	31 (29.8%)	0.458
**Menopausal status**			0.331
Menopausal	55 (52.9%)	48 (46.2%)	
Premenopausal	49 (47.1%)	56 (53.8%)	
**Number of parities**			0.915
None	33 (31.7%)	35 (33.7%)	
1–2	58 (55.8%)	55 (52.9%)	
>2	13 (12.5%)	14 (13.5%)	
**Place of origin**			0.564
Rural	40 (38.5%)	36 (34.6%)	
Urban	64 (61.5%)	68 (65.4%)	
**Occupation**			0.177
Employed	64 (61.5%)	55 (52.9%)	
Unemployment	17 (16.3%)	28 (26.9%)	
Retired	23 (22.1%)	21 (20.2%)	
**Level of income**			0.748
Low	22 (21.2%)	26 (25.0%)	
Medium	58 (55.8%)	53 (51.0%)	
High	24 (23.1%)	25 (24.0%)	
**Civil status**			0.862
Married	87 (83.7%)	84 (80.8%)	
Single	6 (5.8%)	7 (6.7%)	
Divorced/Widowed	11 (10.6%)	13 (12.5%)	
SARS-CoV-2 infection	-	18 (17.3%)	-

* Chi-square or Fisher’s exact test; ** Student’s *t*-test.

**Table 2:** Cervical cancer characteristics of women undergoing radiation therapy before and during the COVID-19 pandemic.

	Before COVID-19 (*n* = 104)	During COVID-19 (*n* = 104)	*p*-Value *
**Comorbidities**			
Hypertension	39 (37.5%)	41 (39.4%)	0.775
Diabetes mellitus	17 (16.3%)	19 (18.3%)	0.713
Ischemic heart disease	9 (8.7%)	11 (10.6%)	0.638
Others	28 (26.9%)	33 (31.7%)	0.446
**Cancer histology**			0.724
Squamous cell carcinoma	85 (81.7%)	83 (79.8%)	
Adenocarcinoma	19 (18.3%)	21 (20.2%)	
**Tumor size**			0.037
<3 cm	59 (56.7%)	44 (42.3%)	
≥3 cm	45 (43.3%)	123 (57.7%)	
**Invasion of vagina**			0.046
Not invaded	23 (22.1%)	15 (14.4%)	
Upper third	41 (39.4%)	30 (28.8%)	
Middle Third	27 (26.0%)	34 (32.7%)	
Lower third	13 (12.5%)	25 (24.0%)	
**Parameters**			0.204
Not invaded	82 (78.8%)	89 (85.6%)	
Invaded	22 (21.2%)	15 (14.4%)	
**Differentiation grade**			0.853
Grade 1	57 (54.8%)	53 (51.0%)	
Grade 2	30 (28.8%)	33 (31.7%)	
Grade 3	17 (16.3%)	18 (17.3%)	
**FIGO stage**			0.032
IB2	11 (10.6%)	6 (5.8%)	
IIA1-IIB	48 (46.2%)	36 (34.6%)	
IIIA-IIIC2	40 (38.5%)	47 (45.2%)	
IVA-IVB	5 (4.8%)	15 (14.4%)	
Radical hysterectomy	24 (23.1%)	29 (27.9%)	0.426
**Relapse**	17 (16.3%)	29 (27.9%)	0.044
Local	8 (47.1%)	9 (31.0%)	0.337
Regional	5 (29.4%)	7 (24.1%)	
Distant	4 (23.5%)	13 (44.8%)	
Palliation	50 (48.1%)	66 (63.5%)	0.025
**Reason for palliation**			0.034
Adjacent organ infiltration	48 (46.2%)	41 (39.4%)	
Distant metastasis	40 (38.5%)	56 (53.8%)	
Poor performance status	16 (15.4%)	7 (6.7%)	
Hospitalization required	26 (25.0%)	33 (31.7%)	0.281
Days of hospitalization	5.1 ± 3.8	6.0 ± 4.1	0.102 **

* Chi-square or Fisher’s exact test; ** Student’s *t*-test.

**Table 3:** Radiotherapy characteristics of women with cervical cancer before and during the COVID-19 pandemic.

	Before COVID-19 (*n* = 104)	During COVID-19 (*n* = 104)	*p*-Value *
**Radiation therapy type**			0.601
External Beam Radiotherapy	82 (78.8%)	85 (81.7%)	
Brachytherapy	22 (21.2%)	19 (18.3%)	
**Moderate/Severe acute toxicity**			
Upper GI	25 (24.0%)	22 (21.2%)	0.618
Lower GI and pelvis	48 (46.2%)	51 (49.0%)	0.677
Genitourinary	21 (20.2%)	26 (25.0%)	0.407
Anemia	63 (64.4%)	69 (66.3%)	0.770
Leucopenia	60 (57.7%)	57 (54.8%)	0.674
Skin toxicity	52 (50.0%)	55 (52.9%)	0.677
**Moderate/Severe late toxicity**			
Bladder	9 (8.7%)	13 (12.5%)	0.367
Intestines	28 (26.9%)	32 (30.8%)	0.540
Kidney	4 (3.8%)	5 (4.8%)	0.733
Skin	2 (1.9%)	0 (0.0%)	0.155
**Response to treatment at 3 months**			0.045
Complete response	82 (78.8%)	77 (74.0%)	
Partial response	10 (9.6%)	4 (3.8%)	
Disease progression	12 (11.5%)	23 (22.1%)	
**Referral source**			0.025
Primary care	67 (64.4%)	51 (49.0%)	
Secondary care	37 (35.6%)	53 (51.0%)	
**Referred to and received treatment**			0.021
Yes	87 (83.7%)	73 (70.2%)	
No	17 (16.3%)	31 (29.8%)	
**Outcomes**			
Change in treatment plan	14 (13.5%)	26 (25.0%)	0.034
Postponed treatment	10 (9.6%)	22 (21.2%)	0.021
Missed appointments	11 (12.5%)	24 (23.1%)	0.015

* Chi-square or Fisher’s exact test.

**Table 4:** Risk factors for disease progression after finishing the radiation therapy regimen.

Risk Factors	HR	CI	*p*-Value
FIGO stage	3.39	2.06–4.21	<0.001
Tumor size	3.12	2.24–4.00	<0.001
Invasion of vagina	2.58	1.82–3.73	<0.001
Postponed treatment	2.51	1.90–3.46	0.001
Missed appointments	2.24	1.18–3.53	0.001
Response to treatment at 3 months	1.66	1.09–2.52	0.014
Age	1.35	1.01–1.84	0.033

FIGO—International Federation of Gynecology and Obstetrics; HR—Hazard Ratio; CI—Confidence Interval.

## Data Availability

The data presented in this study are available on request from the corresponding author.

## References

[1] Castanon A., Tataru D., Sasieni P. (2020). Survival from Cervical Cancer Diagnosed Aged 20–29 Years by Age at First Invitation to Screening in England: Population-Based Study. Cancers.

[2] Daponte A., Michail G., Daponte A.-I., Daponte N., Valasoulis G. (2021). Urine HPV in the Context of Genital and Cervical Cancer Screening—An Update of Current Literature. Cancers.

[3] Sladič M., Taneska P., Cvjetičanin B., Velikonja M., Smrkolj V., Smrkolj Š. (2022). Cervical Intraepithelial Neoplasia Grade 3 in a HPV-Vaccinated Patient: A Case Report. Medicina.

[4] Okayama K., Kimura H., Teruya K., Ishii Y., Fujita K., Fujii M., Oda M., Sasagawa T., Okodo M. (2020). Correlation between Human Papillomavirus Codetection Profiles and Cervical Intraepithelial Neoplasia in Japanese Women. Microorganisms.

[5] Gil-Ibañez B., Gil-Moreno A., Torné A., Martín Jimenez A., Gorostidi M., Zapardiel I., Tejerizo Garcia A., Diaz-Feijoo B., on behalf of SEGO Spain-GOG Cervical Cancer Task Forcé (2022). Tumor Size and Oncological Outcomes in Patients with Early Cervical Cancer Treated by Fertility Preservation Surgery: A Multicenter Retrospective Cohort Study. Cancers.

[6] Popescu A., Craina M., Pantea S., Pirvu C., Radu D., Marincu I., Bratosin F., Bogdan I., Hosin S., Citu C. (2022). COVID-19 Pandemic Impact on Surgical Treatment Methods for Early-Stage Cervical Cancer: A Population-Based Study in Romania. Healthcare.

[7] Margan R., Margan M.-M., Fira-Mladinescu C., Putnoky S., Tuta-Sas I., Bagiu R., Popa Z.L., Bernad E., Ciuca I.M., Bratosin F. (2022). Impact of Stress and Financials on Romanian Infertile Women Accessing Assisted Reproductive Treatment. Int. J. Environ. Res. Public Health.

[8] Tirnea L., Bratosin F., Vidican I., Cerbu B., Turaiche M., Timircan M., Margan M.-M., Marincu I. (2021). The Efficacy of Convalescent Plasma Use in Critically Ill COVID-19 Patients. Medicina.

[9] Dehelean L., Papava I., Musat M.I., Bondrescu M., Bratosin F., Bucatos B.O., Bortun A.-M.C., Mager D.V., Romosan R.S., Romosan A.-M. (2021). Coping Strategies and Stress Related Disorders in Patients with COVID-19. Brain Sci..

[10] Timircan M., Bratosin F., Vidican I., Suciu O., Turaiche M., Bota A.V., Mitrescu S., Marincu I. (2021). Coping Strategies and Health-Related Quality of Life in Pregnant Women with SARS-CoV-2 Infection. Medicina.

[11] Marincu I., Bratosin F., Vidican I., Bostanaru A.-C., Frent S., Cerbu B., Turaiche M., Tirnea L., Timircan M. (2021). Predictive Value of Comorbid Conditions for COVID-19 Mortality. J. Clin. Med..

[12] Timircan M., Bratosin F., Vidican I., Suciu O., Tirnea L., Avram V., Marincu I. (2021). Exploring Pregnancy Outcomes Associated with SARS-CoV-2 Infection. Medicina.

[13] Cerbu B., Pantea S., Bratosin F., Vidican I., Turaiche M., Frent S., Borsi E., Marincu I. (2021). Liver Impairment and Hematological Changes in Patients with Chronic Hepatitis C and COVID-19: A Retrospective Study after One Year of Pandemic. Medicina.

[14] Citu I.M., Citu C., Gorun F., Neamtu R., Motoc A., Burlea B., Rosca O., Bratosin F., Hosin S., Manolescu D. (2022). Using the NYHA Classification as Forecasting Tool for Hospital Readmission and Mortality in Heart Failure Patients with COVID-19. J. Clin. Med..

[15] Citu C., Gorun F., Motoc A., Ratiu A., Gorun O.M., Burlea B., Neagoe O., Citu I.M., Rosca O., Bratosin F. (2022). Evaluation and Comparison of the Predictive Value of 4C Mortality Score, NEWS, and CURB-65 in Poor Outcomes in COVID-19 Patients: A Retrospective Study from a Single Center in Romania. Diagnostics.

[16] Goshen-Lago T., Szwarcwort-Cohen M., Benguigui M., Almog R., Turgeman I., Zaltzman N., Halberthal M., Shaked Y., Ben-Aharon I. (2020). The Potential Role of Immune Alteration in the Cancer–COVID19 Equation—A Prospective Longitudinal Study. Cancers.

[17] Citu I.M., Citu C., Margan M.-M., Craina M., Neamtu R., Gorun O.M., Burlea B., Bratosin F., Rosca O., Grigoras M.L. (2022). Calcium, Magnesium, and Zinc Supplementation during Pregnancy: The Additive Value of Micronutrients on Maternal Immune Response after SARS-CoV-2 Infection. Nutrients.

[18] Seth G., Sethi S., Bhattarai S., Saini G., Singh C.B., Aneja R. (2020). SARS-CoV-2 Infection in Cancer Patients: Effects on Disease Outcomes and Patient Prognosis. Cancers.

[19] Cerbu B., Grigoras M.L., Bratosin F., Bogdan I., Citu C., Bota A.V., Timircan M., Bratu M.L., Levai M.C., Marincu I. (2022). Laboratory Profile of COVID-19 Patients with Hepatitis C-Related Liver Cirrhosis. J. Clin. Med..

[20] Nadarajan G.D., Omar E., Abella B.S., Hoe P.S., Shin S.D., Ma M.H.-M., Ong M.E.H. (2020). A conceptual framework for Emergency department design in a pandemic. Scand. J. Trauma Resusc. Emerg. Med..

[21] Barragán Martín A.B., Molero Jurado M.d.M., Pérez-Fuentes M.d.C., Santillán García A., Jiménez-Rodríguez D., Fernández Martínez E., Herrera-Peco I., Martos Martínez Á., Franco Valenzuela R., Méndez Mateo I. (2021). Adaptation to Change Questionnaire for Nurses: Validation and New Needs in the Context of COVID-19. Healthcare.

[22] Cannedy S., Bergman A., Medich M., Rose D.E., Stockdale S.E. (2022). Health System Resiliency and the COVID-19 Pandemic: A Case Study of a New Nationwide Contingency Staffing Program. Healthcare.

[23] Boilève A., Stoclin A., Barlesi F., Varin F., Suria S., Rieutord A., Blot F., Netzer F., Scotté F. (2020). COVID-19 management in a cancer center: The ICU storm. Support. Care Cancer.

[24] Marincu I., Citu C., Bratosin F., Bogdan I., Timircan M., Gurban C.V., Bota A.V., Braescu L., Grigoras M.L. (2022). Clinical Characteristics and Outcomes of COVID-19 Hospitalized Patients: A Comparison between Complete mRNA Vaccination Profile and Natural Immunity. J. Pers. Med..

[25] Citu I.M., Citu C., Gorun F., Sas I., Tomescu L., Neamtu R., Motoc A., Gorun O.M., Burlea B., Bratosin F. (2022). Immunogenicity Following Administration of BNT162b2 and Ad26.COV2.S COVID-19 Vaccines in the Pregnant Population during the Third Trimester. Viruses.

[26] Davies J.M., Spencer A., Macdonald S., Dobson L., Haydock E., Burton H., Angelopoulos G., Martin-Hirsch P., Wood N.J., Thangavelu A. (2022). Cervical cancer and COVID-an assessment of the initial effect of the pandemic and subsequent projection of impact for women in England: A cohort study. BJOG Int. J. Obstet. Gynaecol..

[27] Perkins R.B., Guido R.S., Castle P.E., Chelmow D., Einstein M.H., Garcia F., Huh W.K., Kim J.J., Moscicki A.-B., Nayar R. (2020). 2019 ASCCP risk-based management consensus guidelines for abnormal cervical cancer screening tests and cancer precursors. 2019 ASCCP Risk-Based Management Consensus Guidelines Committee. J. Low. Genit. Tract Dis..

[28] Grigoraş M.L., Ar-ghirescu T.S., Folescu R., Talpoş I.C., Gîndac C.M., Zamfir C.L., Cornianu M., Anghel M.D., Levai C.M. (2017). Expression of E-cadherin in lung carcinoma, other than those with small cells (NSCLC). Rom. J. Morphol. Embryol..

[29] Chino J., Annunziata C.M., Beriwal S., Bradfield L., Erickson B.A., Fields E.C., Fitch K., Harkenrider M.M., Holschneider C.H., Kamrava M. (2020). Radiation Therapy for Cervical Cancer: Executive Summary of an ASTRO Clinical Practice Guideline. Pract. Radiat. Oncol..

[30] Bhatla N., Berek J., Cuello M., Denny L.A., Grenman S., Karunaratne K. New revised FIGO staging of cervical cancer. Abstract S020.2. Proceedings of the FIGO XXII World Congress of Gynecology and Obstetrics.

[31] Hong K.H., Lee S.W., Kim T.S., Huh H.J., Lee J., Kim S.Y., Park J.-S., Kim G.J., Sung H., Roh K.H. (2020). Guidelines for Laboratory Diagnosis of Coronavirus Disease 2019 (COVID-19) in Korea. Ann. Lab. Med..

[32] Bogdan I., Citu C., Bratosin F., Malita D., Romosan I., Gurban C.V., Bota A.V., Turaiche M., Bratu M.L., Pilut C.N. (2022). The Impact of Multiplex PCR in Diagnosing and Managing Bacterial Infections in COVID-19 Patients Self-Medicated with Antibiotics. Antibiotics.

[33] Popescu A., Craina M., Pantea S., Pirvu C., Chiriac V.D., Marincu I., Bratosin F., Bogdan I., Hosin S., Citu C. (2022). COVID-19 Pandemic Effects on Cervical Cancer Diagnosis and Management: A Population-Based Study in Romania. Diagnostics.

[34] Faye M.D., Alfieri J. (2022). Advances in Radiation Oncology for the Treatment of Cervical Cancer. Curr. Oncol..

[35] Lazzari C., Karachaliou N., Bulotta A., Viganó M., Mirabile A., Brioschi E., Santarpia M., Gianni L., Rosell R., Gregorc V. (2018). Combination of immunotherapy with chemotherapy and radiotherapy in lung cancer: Is this the beginning of the end for cancer?. Ther. Adv. Med. Oncol..

[36] Zhang Y.-F., Fan Y., Zhang P., Ruan J.-Y., Mu Y., Li J.-K. (2022). Cervical Cancer Recurrence and Patient Survival after Radical Hysterectomy Followed by Either Adjuvant Chemotherapy or Adjuvant Radiotherapy with Optional Concurrent Chemotherapy: A Systematic Review and Meta-Analysis. Front. Oncol..

[37] Biplab M., Tapas M., Debarshi L., Sanjoy R., Prabir C., Dilip R.K. (2021). Intensity modulated radiotherapy in carcinoma cervix with metastatic para-aortic nodes: An institutional experience from a Regional Cancer Centre of Eastern India. Rep. Pract. Oncol. Radiother..

[38] Furtunescu F., Bohiltea R.E., Neacsu A., Grigoriu C., Pop C.S., Bacalbasa N., Ducu I., Iordache A.-M., Costea R.V. (2022). Cervical Cancer Mortality in Romania: Trends, Regional and Rural–Urban Inequalities, and Policy Implications. Medicina.

[39] Acampora A., Grossi A., Barbara A., Colamesta V., Causio F.A., Calabrò G.E., Boccia S., de Waure C. (2020). Increasing HPV Vaccination Uptake among Adolescents: A Systematic Review. Int. J. Environ. Res. Public Health.

